# Ipsilateral Traumatic Fractures of the Proximal and Distal Parts of the Humerus (Floating Arm) With Fracture of Distal Clavicle: A Case Report

**DOI:** 10.5812/ircmj.2839

**Published:** 2013-06-05

**Authors:** Mohammad Taghi Peivandi, Hami Ashraf, Omid Shahpari, Zahra Mostafavian, Mehran Azami, Masoud Mirkazemi, Amirreza Fatehi, Nastur Bekhradianpour

**Affiliations:** 1 Department of Orthopedic Surgery, Mashhad Orthopedic and Trauma Research Center, Faculty of Medicine, Mashhad University of Medical Sciences, Mashhad, IR Iran; 2 Orthopedic and Trauma Research Center, Faculty of Medicine, Mashhad University of Medical Sciences, Mashhad, IR Iran; 3 Faculty of Medicine, Mashhad University of Medical Sciences, Mashhad, IR Iran

**Keywords:** Distal Humerus Fracture, Proximal Humerus Fracture, Clavicle Fracture, Floating Arm

## Dear Editor,

**Figure 1.**
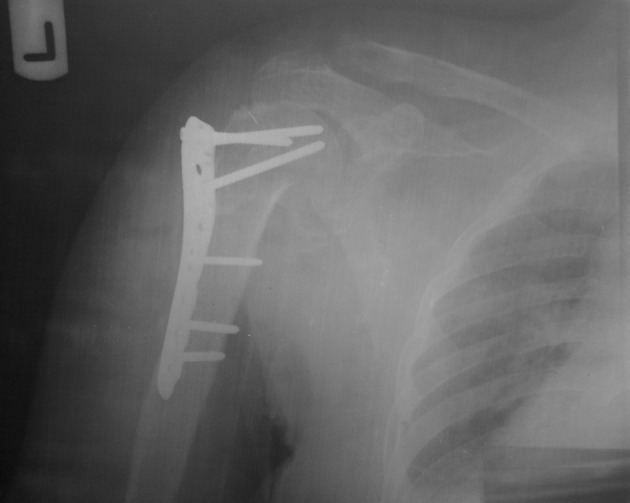
Preoperative Roentgenogram of the Left Proximal Humerus and Distal Clavicle

Asynchronous Non-pathological fractures of proximal and distal parts of humerus and clavicle are common. These are usually the result of traumatic accidents. The prevalence of proximal and distal humerus fractures are 5% and 2% of adult fractures respectively ( 1 , 2 ). Clavicular fractures account for 2.6% of all body fractures. Fractures of the proximal third of the clavicle are uncommon and are less than 5 percent of all clavicles fractures ( 3 , 4 ). Simultaneous Fractures of the proximal and distal parts of the humerus are known as floating arm occurrence of these fractures with clavicle fracture in one limb is very rare. In this study we report a rare case of proximal clavicle fracture with fracture of the proximal and distal parts of the humerus in a 35 years old woman injured from a motor vehicle accident, admitted in Mashhad Shahid Kamyab Hospital. Initially the patient was admitted in the neurosurgery ward, because of head and shoulder trauma. Her radiological study diagnosed left distal clavicular and humerus fracture; hence Velpeu Bandage was done for the patient. After discharge from the neurosurgery ward, she was admitted in the orthopedic ward. Radiography of left elbow was taken because of painful elbow complaints. The X-Ray showed left distal humerus fracture. After three days of her admission, distal humerus fracture was treated with open reduction and internal fixation with reconstruction plate through Triceps Reflecting approach. We used proximal humerus LCP with Deltopectoral approach to fix the proximal humerus fracture. In the middle of the surgery, before going to clavicle, the patient’s general condition was deteriorated, and the operation was stopped by the anesthesiologist. So for distal clavicle fracture, non-operative treatment was recommended. 3 weeks after her discharge from hospital, she started physical therapy and left elbow motions. After a month, other motions of the shoulder and elbow were normal except for 10 degree elbow extension limitation. After 6 months of follow up, the shoulder and elbow range of motion was normal and complete. Union of the distal clavicle was also observed. Distal humerus fracture accompanied with proximal humerus fracture is known as Floating Arm. Cases of distal clavicle fracture along with floating arm in one limb are rarely reported. The prevalence of this kindof fracture is not explained even in references, books or articles. The term floating arm was explained for the first time in an article by Guven et al. on a 10 year old boy. 1 Stanitski and Micheli described floating elbow for the first time in ipsilateral arm and forearm fracture ( 5 ). Gausepohl described floating forearm for ipsilateral elbow and distal radius fracture ( 2 ). The ipsilateral injuries of upper limb can cause some functional limitations in joints, hand or fingers that are important to determine for open or close reduction. Furthermore the coexistence of these uncommon fractures can be missed particularly when the principles of trauma radiology are not attended as seen in this case. The aim of treatment in these patients is to return to demanded functional state. In relevant articles, the optimal treatment for these complex fractures is not mentioned ( 6 - 8 ). The treatment may depend on many factors such as: the health condition of the patient, age, the tolerance for operation and rehabilitation ( 9 ). In the Egol et al study, the result of treatment in 19 patients with floating shoulder showed the outcome of non-operative and operative treatment was similar and desirable ( 10 ). Labler reported that the result of non-operative treatment in patients with minor displacement and operative treatment in patients with major displacement was favorable 8. Themistocleous et al, reported non-operative treatment of ipsilateral fractures of the proximal, midshaft and distal humerus in 80 year old woman with good results ( 9 ). Considering the patient condition we decided to perform open reduction for humerus fractures and conservative treatment for clavicle fracture. In conclusion, the complex humerus fractures are rare and they may be missed. Due to the possibility of injury to the adjacent joints coexisting with the fracture of the relevant limb, routine radiological study of adjacent joints is recommended and satisfactory treatment should be chosen case by case (Figure 1).
